# Study on Assessing Serum Lactate as an Early Prognostic Determinant in Sepsis Outcome

**DOI:** 10.7759/cureus.52186

**Published:** 2024-01-12

**Authors:** Penuboina Tejaswini, Abhishek Singhai, Akash Pawar, Rajnish Joshi, Saurabh Saigal, Abhijit P Pakhare

**Affiliations:** 1 Internal Medicine, All India Institute of Medical Sciences, Bhopal, Bhopal, IND; 2 General Medicine, All India Institute of Medical Sciences, Bhopal, Bhopal, IND; 3 Anesthesiology, All India Institute of Medical Sciences, Bhopal, Bhopal, IND; 4 Community and Family Medicine, All India Institute of Medical Sciences, Bhopal, Bhopal, IND

**Keywords:** severe acute respiratory infection, critical care and internal medicine education, mortality, serum lactate, sepsis

## Abstract

Background: Apart from being one of the main causes of death, sepsis has recently been considered a chronic critical illness. This has resulted in the implementation of standard treatment recommendations for management, with a focus on the initial phases of treatment. Early detection of sepsis and prognostic grading are now crucial for management. Despite the fact that sequential organ failure assessment score (SOFA), acute physiology, and chronic health evaluation II score (APACHE II) have been widely used in sepsis, there have been shortcomings such as feasibility and many lab parameters involved. As a result, this study was conducted to evaluate the role of serum lactate as an early marker and to compare it to current scoring systems for determining the outcome of sepsis.

Methods and Material: This was an observational hospital-based study with 60 individuals recruited over a one-year period from July 2021 to June 2022. Serum lactate, as well as the other laboratory tests required for the computation of SOFA and APACHE II, were performed. The baseline data and the trend of lactate vs standard scores were examined in the first 48 hours, as well as their impact on outcomes in sepsis patients (as measured by mortality rates- patients were followed up for 28 days). The diagnostic accuracy of these scores was calculated using the area under the receiver operating characteristic (ROC) curve (AUROC).

Results: The study enrolled 60 people out of a total of 162 people who were screened. The mean age was 48.4 years, with the highest mortality occurring between the ages of 41 and 60 years. Of the total 60 participants, 34 (56.6%) were male, with the respiratory tract being the most common source of infection for sepsis (36.67%). In our study, 46 patients survived while 14 patients died. The mean lactate on admission was 3.1 mmol/L in survivors and 4 mmol/L in non-survivors, whereas APACHE II was 9 and 12.36, and SOFA was 3.63 and 7.79, respectively, in survivors and non-survivors. Serum lactate and prognosis scores were compared in the survivor and non-survivor groups, and the difference in diagnostic accuracy was found to be statistically significant.

Conclusions: Serum lactate can be used as an early recognition marker in patients with a probability of sepsis and serial lactate monitoring has a similar diagnostic accuracy in predicting outcomes as the traditional prognostic scoring systems SOFA and APACHE II.

## Introduction

Sepsis is defined as life-threatening organ dysfunction caused by an inappropriate host response to infection, and a sequential organ failure assessment (SOFA) score of 2 or above is considered to have organ dysfunction [1]. According to the latest study, approximately 49 million people worldwide are affected with sepsis, with an estimated mortality rate of 20% [2]. In the Southeast Asian region, India has the second highest sepsis burden [3].

Prompt recognition of sepsis and appropriate management in the initial few hours has changed the course and has shown significant improvement in the outcome of the patients. Sepsis is now being reclassified as a chronic critical illness, making early detection and management even more vital [4]. This led to the implementation of one-hour, three-hour, and six-hour sepsis bundles for the earlier treatment of sepsis. After the implementation of these sepsis bundles, it is said to have decreased the in-hospital mortality [5]. It is now even more important to find a means to detect sepsis as soon as possible, for which scoring systems have been applied. The standard prognostic scoring methods for sepsis, SOFA, and acute physiology, and chronic health evaluation II (APACHE II), have a high degree of sensitivity and specificity. One major disadvantage of these scores is that they involve different lab characteristics, and calculating these values is not always feasible. This prompted research into inflammatory marker like lactate, the lactate/albumin ratio, and others.

Initially, serum lactate was assumed to be a byproduct of anaerobic metabolism. Though this notion was correct, recent research proposed an alternative mechanism in which, in sepsis, there may be an increase in lactate levels due to activation of Beta 2 adrenergic receptors and decreased clearance [6]. This approach paved the way for serum lactate to be identified as a signal of impending occult hypoperfusion. This is why high serum lactate levels are included as a perfusion marker in the surviving sepsis guidelines for early goal-oriented therapy [7]. Initial high lactate in the presence of a suspected infection is regarded as a biomarker for sepsis, indicating organ hypoperfusion and tissue hypoxia indirectly. However, studies on serial lactate monitoring as a prognostic marker in sepsis and septic shock patients remain contentious. In our study, we intend to evaluate serum lactate levels at specific time intervals with APACHE II and SOFA scores, as well as their accuracy in predicting study group participant outcomes.

## Materials and methods

This was an observational hospital-based prospective cohort study. The study was initiated after the design and protocol had been approved by the Institutional Human Ethics Committee of AIIMS Bhopal (Reference no. 2020/PG/July/17 dated February 27, 2021). All the study participants underwent study procedures after a written informed consent.

We approached 162 individuals over the age of 18 who had a suspected or diagnosed case of sepsis and a SOFA score of 2 or higher for possible participation in our study. The Third International Consensus Definitions for Sepsis and Septic Shock (Sepsis-3) defined sepsis as life-threatening organ dysfunction caused by a dysregulated host response to infection. Fifty-four participants were excluded from the study for a variety of reasons, including a time-lapse of more than six hours between patient recognition, being on inotrope support at the time of recruitment, and having comorbidities such as ischemic cardiomyopathy, cancer, liver failure, or seizure disorder.

**Figure 1:**
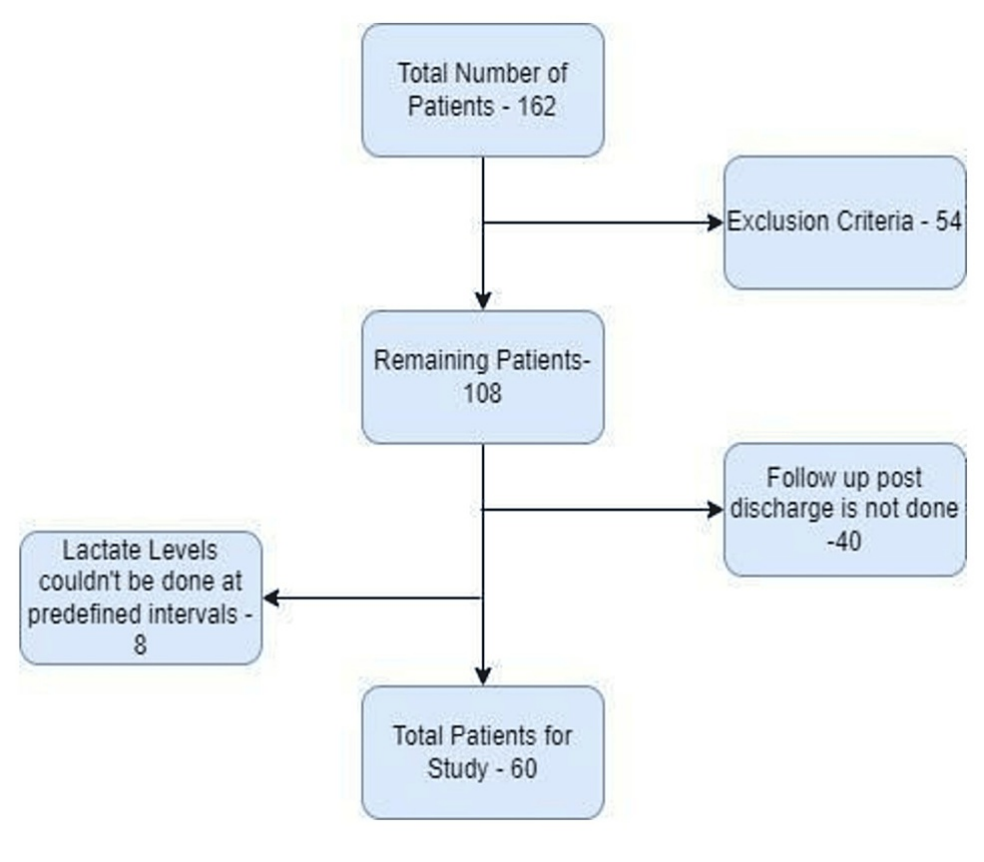
Flow chart depicting the enrolment of the study population

The study included 108 participants after excluding 54 individuals. We followed up with the patient for one month after he/she was discharged from the hospital. Forty participants were lost to follow-up during the study period, and eight people's serum lactate levels could not be monitored at the prescribed intervals; thus, they were also dropped. As a result, 60 participants' data were evaluated for outcomes (Figure 1).

Demographic information, clinical findings, laboratory results, the presence of comorbidities, and the need for inotrope support were all obtained. Complete blood count, liver function test, and renal function test data were taken at baseline on admission or when sepsis was recognized, and the same investigations were repeated after 48 hours. Serum lactate levels were measured at the start, 12 hours, and 48 hours. Finally, based on the lab values available at baseline and 48 hours, SOFA and APACHE II scores were determined. The primary outcome of the study was to study the role of lactate and its accuracy as the early prognostic determinant in the outcome of sepsis. Secondary outcomes were to compare the accuracy of serial lactate measurements with that of APACHE II and SOFA in prognostication and follow-up of the patients for the next 28 days for outcome.

Statistical analysis

The data obtained throughout were ﬁnally analyzed using the SPSS software version 21 (IBM Corp., Armonk, NY). We used the chi-square test for dichotomous variables and the student's t-test or Wilcoxon rank-sum test for continuous variables to compare proportions, means, or medians, respectively. Confounding comorbidities were analyzed by appropriate univariate/ multivariate analysis.

We considered a p-value of <0.05 as a signiﬁcance level for these comparisons. Area under receiver operating characteristic (ROC) curves (AUROC) have been used to approximate the estimation for diagnostic accuracy between the standard prognostic scores and the serum lactate levels.

## Results

The study enrolled 60 of the 162 people who were screened. There were 34 (56.7%) males and 26 (43.3%) females among the 60 participants. Individuals with no comorbidities account for 31 (51.67%), whereas the remainder have one or more comorbidities. Lower respiratory tract infection (35%) was the most common cause of sepsis in the study group population, followed by urinary tract infection (21.67%) (Table 1).

**Table 1:** Demographic details of the patients recruited in the study

Variable	N (%)
Age in years	
Age in year (Mean ± SD)	48.4 ± 19.9
Range	18-87
Gender	
Male	34 (56.7%)
(Male age in year (Mean ± SD)	50.6 ± 20.6
Female	26 (43.3%)
Female age in year (Mean ± SD)	45.5 ± 19.8
Source of infection	
Blood Stream Infection	1 (1.67%)
Skin soft tissue infection	2 (3.34%)
Gastrointestinal sepsis	6 (4%)
Lower respiratory tract infection	21 (35%)
Lower respiratory tract infection and urinary tract infection	1 (1.67%)
Urinary tract infection	13 (21.67%)
Others	19 (31.67%)
No comorbidities	31 (51.67%)
1 comorbidity	25 (41.67%)
≥2 comorbidities	4 (6.67%)

Further features of the study participants were examined in two groups: survivors and non-survivors (46 and 14 persons, respectively) (Table 2). Univariate analysis was performed, taking into account the individual lab data needed to calculate the prognostic scores SOFA and APACHE II. Some of the important lab parameters taken for analysis are mentioned.

**Table 2:** Lab parameters and scores in the study group at baseline and at 48 hours

Variable	Survivor (N= 46) (+SD)	Non-survivor (N= 14) (+SD)	P-value
P/F Ratio at diagnosis	370 (64.3)	346 (63.5)	0.109
P/F Ratio at 48 hours	341 (98)	226 (115)	0.002
Serum sodium at diagnosis (mEq/L)	133 (7.33)	136 (14.84)	0.889
Serum sodium at 48 hours (mEq/L)	135.45 (7.74)	135.7 (10.95)	0.528
Serum creatinine at diagnosis (mg/dl)	1.55 (0.82)	1.23 (0.62)	0.202
Serum creatinine at 48 hours (mg/dl)	1.55 (1.19)	1.98 (1.47)	0.576
pH at diagnosis	7.35 (0.12)	7.36 (0.09)	0.643
pH at 48 hours	7.37 (0.05)	7.39 (0.07)	0.681
Platelet count at diagnosis	1.84 L (1.03)	2.23 (1.13)	0.128
Platelet count at 48 hours	1.70 L (1.03)	1.92L (1.28)	0.773
GCS at diagnosis	14.8 (0.65)	14.5 (1.16)	0.051
GCS at 48 hours	14.7 (0.88)	12.6 (2.07)	<0.001
Prognostic Scores and Lactate at Baseline and at 48 Hours			
SOFA at diagnosis	2	2	
SOFA at 48 hours	3.63 (3.38)	7.79 (2.61)	<0.001
APACHE II at diagnosis	9.93 (5.4)	12.36 (3.37)	0.103
APACHE II at 48 hours	10.24 (7.88)	17.64 (5.92)	0.002
Serum lactate at diagnosis (mmol/L)	3.1 (2.2-3.9)	4 (3.6-5.1)	0.003
Serum lactate at 12 hours (mmol/L)	2.4 (1.8-3.8)	4.0 (3.3-5.1)	<0.001
Serum lactate at 48 hours (mmol/L)	2.1 (1.2-3.1)	4 (4.1-5.9)	<0.001

As shown in Table 2, serum lactate, even at diagnosis, has been shown to be statistically significant in predicting the outcome, i.e., mortality, and so might be considered as a single indicator that could be employed in the early detection of sepsis.

Among the parameters measured 48 hours after admission or diagnosis, the individual values of arterial pO2/FIO2 (P/F) ratio and Glasgow coma scale (GCS) help determine the outcome in sepsis as time progresses. The decrease in GCS and P/F ratio from admission to 48 hours was statistically significant among survivors and non-survivors.

According to earlier research, APACHE II and SOFA are effective in assessing the prognosis of sepsis, and this is reflected in our study, which had a p-value of 0.005, which was statistically significant. However, the baseline serum lactate value and trend in serum lactate are both statistically significant and could be utilized as prognostic markers in sepsis.

**Figure 2:**
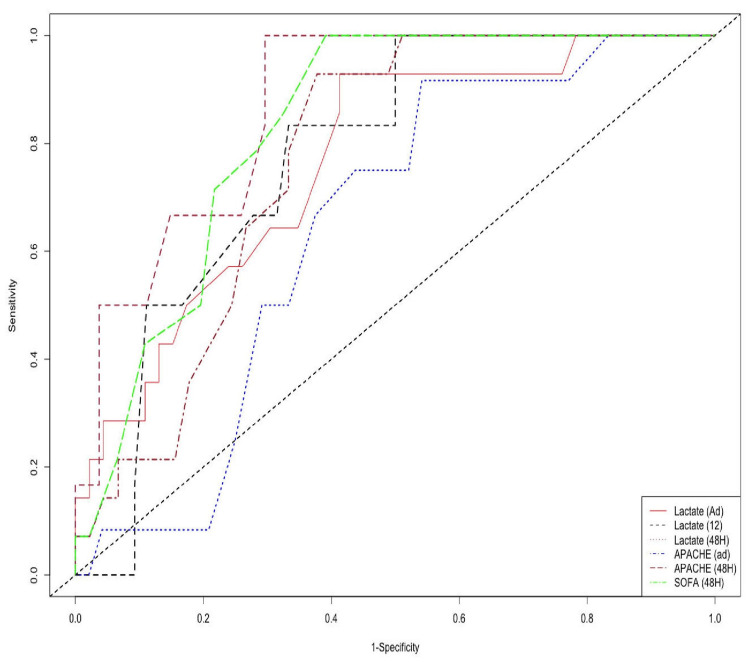
Time-dependent ROC for predicting outcomes (day 28) The ROC curves of SOFA, APACHE II and Serum lactate at 48 hours depict that there has been a comparable sensitivity pattern in predicting the outcomes in sepsis with diagnostic accuracy almost similar between lactate and the scoring systems.

Although the serum lactate trend has been shown to be statistically significant in predicting prognosis in our study, the accuracy of this trend is compared with the existing prognostic grading systems APACHE II and SOFA utilizing ROC curves (Table 3). The AUROC percentages of our study group population who were observed for up to 28 days after admission or diagnosis are shown in Figure 2. It has been noted that, at any given time, both admission lactate and serial lactate levels are as effective as APACHE II and SOFA scores in predicting sepsis outcomes.

**Table 3:** Area under ROC curve value in percentages for predicting the mortality at diﬀerent times

Day	Serum lactate (Admission)	Serum lactate (12 hours)	Serum lactate (48 hours)	APACHE II (Admission)	APACHE II (48 hours)	SOFA (48 hours)
t =7	59.3	77.3	89.8	52.0	71.7	81.3
t =14	65.4	77.6	87.0	56.9	68.6	80.2
t =21	75.3	76.7	91.8	64.3	75.3	84.6
t =28	76.6	80.4	92.9	64.5	77.1	82.9

## Discussion

SOFA and APACHE II scores have been used as prognostic scoring systems in septic patients for decades. However, due to laboratory constraints, numerous investigations were conducted in order to determine the simplest and best prognostic marker. Serum lactate measurements have so taken on a more significant role in recent years. Despite the emphasis on lactate monitoring, there is currently a dearth of evidence proving that initial and serial lactate monitoring can result in better results [8].

There were 60 participants in our study, with a mean age of 48.4 and a majority of the individuals fell between the ages of 41 and 60. Our study had a male preponderance, with 56.6% of the participants being male compared to 43.3% being female. This study's findings were comparable to those of Innocenti et al., who found a male prevalence of 59% [9]. We found that respiratory tract infections were the most common source of infection for sepsis, followed by urinary tract infections. In this study, we examined a large number of univariate variables, which are the individual components employed in scoring system evaluation. However, all the variables turned out to be statistically insigniﬁcant except for the two components which are GCS and P/F ratio at baseline and 48 hours post admission. This depicts that rather than a single value the change in these values with time has a significant role in assessing the outcomes of sepsis.

Chen-yang et al. did a study on sepsis patients that found comparable findings to ours for GCS values, showing that mental confusion was present in 46% of non-survivors and 20% of survivors [10]. Mikkelsen et al. demonstrated that a P/F ratio of 300 in severe sepsis accounted for just 11.3% of persons but that serum lactate and P/F ratio had a significant correlation with mortality [11].

According to our study, 14 patients died within 28 days of the follow-up period, accounting for the non-survivor group, while the remaining 46 people were survivors. Mean lactate scores on admission were 3.1 mmol/L (2.2-3.9) in the survivor group and 4 mmol/L (3.6-5.1) in the non-survivor group and were found to be statistically significant with a p-value of <0.005. The change, i.e., rises and falls in the mean serum lactate levels has been found in the non-survivor and survivor groups, respectively, which are also statistically significant.

Nada et al. conducted a study on sepsis patients with pneumonia as the source of infection, and observations on serum lactate revealed values of 4.9 mmol/L with a standard deviation of 3.23 in survivors and 7.1 mmol/L with a standard deviation of 3.61 in non survivors. These findings matched those of our study [12]. Husain et al. conducted research in surgical ICU patients on lactate clearance and found that normalization of lactate after 24 hours reduced mortality to 23% when the initial lactate was 3.1 [13]. These findings were essentially identical to our study, where a 48-hour decrease in serum lactate reduced mortality rates to 23.3% when the starting mean serum lactate was 3.1 mmol/L.

In the multivariate analysis, we found comparability and accuracy between serum lactate, APACHE II, and SOFA in predicting sepsis outcomes. The admission mean APACHE II in the survivor and non-survivor groups was 9.93 and 12.36, respectively, whereas the 48-hour mean was 10.24 and 17.64, which is statistically significant. The admission SOFA score (inclusion criterion of 2) was the same in all 60 individuals, and the 48-hour SOFA was 3.63 and 7.79 in the survivor and non-survivor groups, respectively. The admission mean lactate in the survivor and non-survivor groups was 3.1 mmol/L and 4 mmol/L, respectively, while the 48-hour mean lactate was 2.1 mmol/L and 5.2 mmol/L, all of which were statistically significant. Murat et al. described similar findings of these three scores, which showed that mean lactate in survivors and non-survivors were 2 and 3, respectively, mean APACHE II was 19 and 30 in respective groups, and mean SOFA scores were 5 and 7 in respective subgroups, all of which were statistically significant in assessing outcome in sepsis [14]. The AUROC for lactate, APACHE II, and SOFA scores and their percentages at any point in time until day 28 of follow-up from diagnosis demonstrated that changes in lactate values have similar results. Also, a single serum lactate value was shown to have a slightly greater percentage than the other two scores, indicating that serum lactate could be the single best signal for early detection and prognosis of sepsis. Chaudhari et al. conducted a similar study and concluded that higher SOFA, qSOFA, and serum lactate levels were linked to a higher 28th-day mortality rate. Poor outcomes were also linked to low absolute, relative, and rate of lactate clearance. The best cutoffs for predicting bad outcomes were relative lactate clearance of 40.3% and serum lactate level at 24 hours 4 mmol/L, both of which had good sensitivity and specificity [15]. Nabhat et al. showed that the high blood lactate (≥2 mmol/L) group had significantly higher 28 days mortality (31.9% vs 10.0%; p < 0.001) and subsequent three days septic shock (18.1% vs 5.0%; p < 0.001) than the normal blood lactate group whereas in our study non-survivor group (23.3%) had initial lactate more than 3.6 mmol/L [16]. Jaiswal et al. had also done a similar study and concluded that in comparison with SOFA and APACHE IV scores, serial serum lactate was reliable and comparable as far as an outcome in terms of mortality is concerned in patients with sepsis admitted to the intensive care unit [17].

Our study has some limitations including a small sample size and the exclusion of some individuals with comorbidities, which may have influenced the study's findings.

## Conclusions

When there is a suspicion of sepsis, serum lactate can be utilized as an early determinant (exceptions include comorbidities and medicines that may act as confounding factors). In sepsis patients, serum lactate can be used as a single best prognostic indicator, and its diagnostic accuracy is comparable to the other prognostic scores SOFA and APACHE II.

## References

[1] Singer M, Deutschman CS, Seymour CW (2016). The Third International Consensus Definitions for sepsis and septic shock (Sepsis-3). JAMA.

[2] (2023). Sepsis in India Prevalence Study (SIPS). https://www.georgeinstitute.org.in/projects/sepsis-in-india-prevalence-study-sips.

[3] Mehta Y, Paul R, Rabbani R, Acharya SP, Withanaarachchi UK (2022). Sepsis management in Southeast Asia: a review and clinical experience. J Clin Med.

[4] Hotchkiss RS, Moldawer LL, Opal SM, Reinhart K, Turnbull IR, Vincent JL (2016). Sepsis and septic shock. Nat Rev Dis Primers.

[5] Dugar S, Choudhary C, Duggal A (2020). Sepsis and septic shock: guideline-based management. Cleve Clin J Med.

[6] Levy B, Desebbe O, Montemont C, Gibot S (2008). Increased aerobic glycolysis through beta2 stimulation is a common mechanism involved in lactate formation during shock states. Shock.

[7] Ding XF, Yang ZY, Xu ZT (2018). Early goal-directed and lactate-guided therapy in adult patients with severe sepsis and septic shock: a meta-analysis of randomized controlled trials. J Transl Med.

[8] Basile-Filho A, Lago AF, Menegueti MG (2019). The use of APACHE II, SOFA, SAPS 3, C-reactive protein/albumin ratio, and lactate to predict mortality of surgical critically ill patients: a retrospective cohort study. Medicine (Baltimore).

[9] Innocenti F, Meo F, Giacomelli I (2019). Prognostic value of serial lactate levels in septic patients with and without shock. Intern Emerg Med.

[10] Zhang Z, Xu X (2014). Lactate clearance is a useful biomarker for the prediction of all-cause mortality in critically ill patients: a systematic review and meta-analysis. Crit Care Med.

[11] Mikkelsen ME, Miltiades AN, Gaieski DF (2009). Serum lactate is associated with mortality in severe sepsis independent of organ failure and shock. Crit Care Med.

[12] Mohamed N, Mohsen M, Hameed SA, Saad A (2022). Study between lactate clearance and APACHE II score as predictor prognosis of septic patients. Al-Azhar Int Med J.

[13] Chen YX, Li CS (2015). Lactate on emergency department arrival as a predictor of mortality and site-of-care in pneumonia patients: a cohort study. Thorax.

[14] Erdoğan M, Findikli HA (2022). Prognostic value of the lactate/albumin ratio for predicting mortality in patients with pneumosepsis in intensive care units. Medicine (Baltimore).

[15] Chaudhari M, Agarwal N (2022). Study of significance of serum lactate kinetics in sepsis as mortality predictor. Indian J Crit Care Med.

[16] Noparatkailas N, Inchai J, Deesomchok A (2023). Blood Lactate Level and the Predictor of Death in Non-shock Septic Patients. Indian J Crit Care Med.

[17] Jaiswal P, Agrawal S, Kumar S (2022). A two-year cross-sectional study on the impact of serial serum Lactate in comparison with APACHE IV and SOFA Scores in predicting outcomes in patients of sepsis at limited resources rural setup. J Emerg Med Trauma Acute Care.

